# Network Based Scoring Models to Improve Credit Risk Management in Peer to Peer Lending Platforms

**DOI:** 10.3389/frai.2019.00003

**Published:** 2019-05-24

**Authors:** Paolo Giudici, Branka Hadji-Misheva, Alessandro Spelta

**Affiliations:** ^1^Department of Economics and Management, Fintech Laboratory, University of Pavia, Pavia, Italy; ^2^School of Engineering, Zurich University of Applied Sciences (ZHAW), Winterthur, Switzerland

**Keywords:** contagion, credit risk, credit scoring, network models, peer to peer lending

## Abstract

Financial intermediation has changed extensively over the course of the last two decades. One of the most significant change has been the emergence of FinTech. In the context of credit services, fintech peer to peer lenders have introduced many opportunities, among which improved speed, better customer experience, and reduced costs. However, peer-to-peer lending platforms lead to higher risks, among which higher credit risk: not owned by the lenders, and systemic risks: due to the high interconnectedness among borrowers generated by the platform. This calls for new and more accurate credit risk models to protect consumers and preserve financial stability. In this paper we propose to enhance credit risk accuracy of peer-to-peer platforms by leveraging topological information embedded into similarity networks, derived from borrowers' financial information. Topological coefficients describing borrowers' importance and community structures are employed as additional explanatory variables, leading to an improved predictive performance of credit scoring models.

## 1. Introduction

Financial intermediation has changed extensively over the course of the last two decades mostly due to technological advancement. One of the most significant change has been the emergence of FinTech that is nowadays altering many financial products, services, production processes, and organizational structure. In the context of commercial credit, FinTech solutions have introduced many opportunities for both lenders and borrowers thus redefining the role of traditional intermediaries. Peer-to-peer lending platforms, often abbreviated P2P lending, allow private individuals to directly run small and, in most cases, unsecured loans to private borrowers or small and medium enterprises (SME). The recent advances in information technology have enabled these online platforms to provide an alternative to traditional financial intermediaries, by delivering more cost efficient, consumer friendly and transparent lending services, improving the overall value for customers (for a review see e.g., Claessens et al., 2018; Giudici and Misheva, 2018).

The literature identifies many factors which explain the increasing role of P2P lending platforms in the global world of finance (see e.g., Serrano-Cinca and Gutiérrez-Nieto, 2016). For instance, P2P platforms are not required to respect bank capital requirements nor to pay fees associated with state deposit insurance practices, and this allows them to operate with lower costs. Thus, borrowers benefit because they are able to receive credits at lower interest rates, and in some cases with little or no collateral, whereas lenders can receive higher rates of return on investment, due to reduced transaction costs (see Emekter et al., 2015). Second, advancements in information technology have also been a key force driving the exponential growth of P2P platforms (see Guegan and Hassani, 2017). In this context, many P2P platforms rely not only on "hard" but also on "soft" i.e., social network activity information for the purpose of evaluating a candidate's creditworthiness, a practice not typically employed by traditional banks. The third factor explaining the rapid growth of P2P platforms is related with regulatory aspects. With the new revised Payment Service Directive (PSD2), that came in effect in 2018, the monopoly which banks have on their clients account information and payment transactions becomes weaker as this information can be disclosed through application payment interfaces. From a different viewpoint, the rapid growth of the importance of P2P lending platforms can pose significant risks to financial stability. This because P2P lenders typically produce inadequate measures of credit risk. In comparison with traditional banks, P2P platforms are less able to eliminate asymmetric information, thus increasing the risk of bad debt accumulation because they have no access to detailed information on borrowers past financial transaction.

Moreover, P2P lending activity is built on the basis of a "many-to-many" approach, in which the financial intermediary empowers each lender to decide to whom borrower to lend and for what amount. This leads to a strong interdependence between the borrowers and the lenders, which may generate high levels of contagion and systemic risk.

Even more importantly, P2P lenders allow for direct matching between borrowers and lenders, without the loans being held on the intermediary's balance-sheet; in other words, in a P2P platform, the risk is fully born by the lender. From a risk-return perspective, while in classical banking a financial institution chooses its optimal trade-off between risks and returns (subject to regulation constraints), in P2P lending, the platform maximizes its returns without taking care of the risks which are borne by the lenders.

The misaligned incentives, asymmetric information, differences in the business model and in the risk ownership may lead to the platform not being able to correctly distinguish between different risk classes which in turn can impact the overall stability of the financial system. In this paper we propose to exploit topological information embedded into similarity networks to increase the predictive performance of some credit scoring models.

Understanding the structure of a similarity network (see Mantegna and Stanley, 1999) is indeed instrumental for understand the origin of companies failures and to inform policymakers on how to prepare for, and recover from, adverse shocks hitting the network. Similarity patterns between companies' features can be extracted from a distance matrix and they can reveal how credit risk is related to the topology of the network. To account for such topological information we rely on centrality measures and community structure detection (see e.g., Newman, 2018). We show that the inclusion of these variables into credit scoring models does improve their predictive utility. Results confirm the validity of this approach in discriminating between defaulted and sound institutions, thus, the proposed methodology can constitute a new instrument in both policy-makers an practitioners toolboxes. We remark that our work is related to two main other recent research streams. First, some authors have carried out investigations on the accuracy of credit scoring models of P2P platforms (Serrano-Cinca et al., 2016). We improve these contributions by extending the methodology to also account for the interconnections that emerge between economic agents. Second, our network approach relates to a recent and fast expanding line of research which focuses on the application of network analysis tools, for the purpose of understanding flows in financial markets, as in the papers of Allen and Gale (2000), Leitner (2005), and Giudici and Spelta (2016). We improve these contributions, extending them to the P2P context and linking network models, that are often merely descriptive, with statistical and machine learning models, thus providing a predictive framework. The rest of the paper is organized as follows: section 2 introduces the data set we employ in the analysis together with the description of the credit scoring models and of the performance measures. In this section we also present the metric used for extracting distances between the borrowing companies and the methods employed for building the networks and for extracting topological information. Section 3 is devoted to show the results of the analysis and the comparison between the performances of the credit scoring models with and without the topological information. Section 4 concludes.

## 2. Data and Methodology

In this section we first describe the data set employed in our analysis and the necessary pre-processing stage. Subsequently we introduce the families of credit scoring models and the non-parametric measures used for testing the performance of such models. Then we focus on showing how one can extract relevant patterns of similarities to build up meaningful networks from balance-sheet features of borrowing companies.

We consider data supplied by the European External Credit Assessment Institution (ECAI) that specializes in credit scoring for P2P platforms focused on SME commercial lending. Specifically, the analysis relies on a data set, that is composed of official financial information (financial ratios constructed on the basis balance sheet and income statement information) on 4514 Italian SMEs which represent the target of P2P lending platforms. Appendix A provides a table encompassing formulas to compute such ratios. Table 2, instead, provides the summary statistics of the variables included in this data set and information concerning their mean value aggregated by the status of the companies (active and defaulted). It is important to note that none of the variables included in data set contains missing values and the proportion of defaulted companies is 11%.

What is noticeable from Table 1, is that, as in most real-world data sets (and particularly those reflecting the operations of start-ups and small and medium enterprises), for most variables, there is a noticeable presence of unusually large or small values when compared to the mean. The literature recognizes many methods for dealing with outliers however in most cases the correct application of these methods is based on very strong assumptions concerning the size and distribution of the data set as well as the randomness of the outliers. In this context, we do not substitute or cancel outliers because we believe they can provide important insights concerning the companies included in the sample. All data and code employed is available as Supplementary Material.

**Table 1:** Summary statistics of variables included in the dataset.

**Statistic**	***N***	**Mean**	**St. Dev**.	**Min**	**Pctl(25)**	**Pctl(75)**	**Max**	**Active**	**Default**
ratio001	4,514	8.885	19.155	−64.430	1.303	9.680	206.550	8.85	9.15
ratio002	4,514	1.264	3.333	−10	0	1.2	33	1.25	1.35
ratio003	4,514	1.444	0.761	0.170	1.070	1.520	8.270	1.49	1.09
ratio004	4,514	1.536	1.201	0.010	0.970	1.720	13.710	1.6	1.04
ratio005	4,514	1.190	1.024	0.000	0.610	1.407	10.880	1.24	0.76
ratio006	4,514	7.726	23.277	−33.140	0.940	4.890	297.020	7.93	6.09
ratio008	4,514	23.068	70.271	−285.860	1.240	16.317	566.960	26.22	–2.33
ratio011	4,514	0.028	0.147	−1	0.01	0.1	0	0.05	–0.13
ratio012	4,514	−0.069	0.790	−8.540	0.000	0.210	1.080	0.01	–0.69
ratio017	4,514	1.372	1.068	0.010	0.680	1.740	8.420	1.38	1.30
ratio018	4,514	1.335	1.064	0.010	0.640	1.700	8.420	1.34	1.29
ratio019	4,514	0.194	0.498	−3.320	0.010	0.390	3.950	0.21	0.05
ratio027	4,514	36.513	92.893	−191.630	2.470	27.608	747.010	40.18	6.96
ratio029	4,514	0.062	0.196	−2	0.02	0.1	1	0.08	–0.12
ratio030	4,514	0.068	0.216	−2	0.02	0.1	1	0.09	–0.12
DIO	4,514	105.228	355.807	0	1	80	5.569	100.61	142.47
DPO	4,514	75.934	111.651	0	0	99.8	1.467	67.35	145.18
DSO	4,514	95.732	128.370	0	0	136	1.465	91.07	133.32
turnover	4,514	3,344.479	7,580.559	6	594	2,761.8	76.403	3,542.27	1,749.41

*For each measure we report the average (Mean) along with the standard deviation (St. Dev.), the minimum (Min), the 25-th and 75-th percentiles (Pctl), the maximum (Max), mean value of the variable for active companies (Active), mean value of the variable for defaulted companies (Defaulted)*.

### 2.1. Credit Risk Models

Credit risk models are useful tools for modeling and predicting individual firm default. Such models are usually grounded on regression techniques or machine learning approaches often employed for financial analysis and decision-making tasks (see Khandani et al., 2010; Yu et al., 2010; Khashman, 2011; Lessmann et al., 2015; Abellán and Castellano, 2017 to cite few).

Consider *N* firms having observation regarding *T* different variables (usually balance-sheet measures or financial ratios). For each institution *n* define a variable γ_*n*_ to indicate whether such institution has defaulted on its loans or not, i.e., γ_*n*_ = 1 if company defaults, γ_*n*_ = 0 otherwise. In a nutshell, credit risk models develop relationships between the explanatory variables embedded in *T* and the dependent variable γ.

Against this background, we employ logistic regression, discriminant analysis, classification and regression trees and support vector machine (Anderson, 2007). The following paragraphs briefly summarize the characteristics of the models we use for the present analysis.

The logistic regression model is one of the most widely used method for credit scoring. The model aims at classifying the dependent variable into two groups characterized by different status (defaulted v.s. active) by the following model:

(1) $$\begin{array}{r} ln\left(\frac{p_{n}}{1-p_{n}}\right)=\alpha +\overset{T}{\underset{t=1}{\sum }}\beta_{t}x_{nt} \end{array}$$

where *p*_*n*_ is the probability of default for institution *n*, **x**_*i*_ = (*x*_*i*,1_, …, *x*_*i,T*_) is the *T*-dimensional vector of borrower specific explanatory variables, the parameter α is the model intercept while β_*t*_ is the *t*-th regression coefficient. It follows that the probability of default can be found as:

(2) $$\begin{array}{r} p_{n}=(1+exp\left(\alpha +\overset{T}{\underset{t=1}{\sum }}\beta_{t}x_{nt}\right){)}^{-1} \end{array}$$

Discriminant analysis assumes that different classes generate data based on different Gaussian distributions. Linear discriminant analysis (LDA) approaches the problem by assuming that the conditional probability density functions *p*(**x**|γ = 0) and *p*(**x**|γ = 1) are both normally distributed with mean and covariance parameters (*μ*_0_, **V**_0_) and (*μ*_1_, **V**_0_) respectively. In this context, the decision rule is based on the Linear Score Function, a function of the population means for each of the populations, i, as well as the pooled variance-covariance matrix.

Classification and regression trees (CART) is another widely used statistical technique in which a dependent variable is associated with a set of input factors through a recursive sequence of simple binary relations. Put simply, it is a step-by-step process which results in a decision tree which is constructed either by splitting or not splitting each node into daughter nodes. The splitting strategy follows a node impurity function meaning that at each stage of the recursive partitioning, all possibles splits are considered and the one which leads to the greatest increase in node purity is chosen.

Support vector machine (SVM) classifies data by detecting the best hyperplane that separates all data points of one class from those of the other class. Given a data set of *N* institutions of the form (**x**_1_, γ_1_), …, (**x**_*N*_, γ_*N*_) where the γ_*n*_ indicates the class to which the point **x**_*n*_ belongs. Each **x**_*n*_ is a *T*-dimensional real vector. SVM finds the “maximum-margin hyperplane” that separates data points **x**_*n*_ for which γ = 1 from the data points for which γ = 0, which is defined so that the distance between the hyperplane and the nearest point **x**_*n*_ from either group is maximized. In formula:

(3) $$\begin{array}{r} \underset{\text{w}\in R^{T},b\in R}{\max}\underset{\text{x}\in A\cup B}{\min}\frac{\left|{\text{w}}^{\prime }{\text{x}}_{i+b}\right|}{\left||\text{w}|\right|} \end{array}$$

where *A* and *B* are disjoint subsets and **wx** − *b* = 0 represents a hyperplane.

### 2.2. Assessing Model Performance

For evaluating the performance of each model, we employ, as a reference measure, the indicator γ ∈ {0, 1} that is a binary variable which takes value one whenever the institutions has defaulted and value zero otherwise. For detecting default events represented in γ, we need a continuous measurement *p* ∈ [0, 1] to be turned into a binary prediction *B* assuming value one if *p* exceeds a specified threshold τ ∈ [0, 1] and value zero otherwise. The correspondence between the prediction *B* and the ideal leading indicator γ can then be summarized in a so-called confusion matrix.

From the confusion matrix we can easy illustrate the performance capabilities of a binary classifier system. To this aim, we compute the receiver operating characteristic (ROC) curve and the corresponding area under the curve (AUC) and Gini coefficient. The ROC curve plots the false positive rate (FPR) against the true positive rate (TPR). To be more explicit:

(4) $$\begin{array}{r} FPR=\frac{FP}{FP+TN} \end{array}$$

(5) $$\begin{array}{r} TPR=\frac{TP}{TP+FN} \end{array}$$

Moreover, we also compute other measures for assessing models performance such as the accuracy and the KS statistic. The overall accuracy of each model can be computed as:

(6) $$\begin{array}{r} ACC=\frac{TP+TN}{TP+TN+FP+FN} \end{array}$$

and it characterizes the proportion of true results (both true positives and true negatives) among the total number of cases under examination. In this context a key issue is setting the threshold at which a company is classified as belonging to one class rather than another.

Additional to this, another often-used characteristic in describing the quality of the model (or the scoring function) is the Kolmogorov-Smirnov statistic (KS). This metric too seeks to jointly consider specificity and sensitivity and it corresponds to the maximum value of their sum as the threshold is varied. Put differently, it represent the maximum difference between the cumulative distribution of active and defaulted companies. Consequently, the KS statistics is defined as:

$$\begin{array}{l} KS=max_{j}|F_{Active}\left(x_{j}\right)-F_{Defaulted}\left(x_{j}\right)| \end{array}$$

For back-testing, while assessing the performance of each model, available information must be exploited in a realistic manner. To this end, we perform repeated sub-sampling validation approach. Specifically, we randomly split the data set in 10 training and validations data sets. For each such split, the model is fitted on the training data set and predictive utility is assessed on the corresponding testing data. The results concerning the model accuracy (area under the ROC curve, KS statistic, Gini index) are then averaged over the splits.

### 2.3. The Distance Metric

In the present study we exploit information derived from financial statements of borrowing companies collected in a vector **x**_*n*_ representing the financial composition of the balance-sheet of institution *n*. We define a metric that provides the relative distance between companies by applying the standardized Euclidean distance between each pair (**x**_*i*_, **x**_*j*_) of institutions feature vectors. More formally, we define the pairwise distance *d*_*i,j*_ as:

(7) $$\begin{array}{r} d_{i,j}=\left({\text{x}}_{i}-{\text{x}}_{j}\right)\Delta^{-1}{\left({\text{x}}_{i}-{\text{x}}_{j}\right)}^{\prime } \end{array}$$

where **Δ** is a diagonal matrix whose *i*-th diagonal element represent the standard deviation of the series. Namely, each coordinate difference between pairs of vectors (**x**_*i*_−**x**_*j*_) is scaled by dividing by the corresponding element of the standard deviation. The distances can be embedded into a *N* × *N* dissimilarity matrix **D** such that the closer the companies *i,j* features are in the Euclidean space, the lower the entry *d*_*i,j*_.

Although **D** can be informative about the distribution of the distances between the companies, the fully-connected nature of this set does not help to find out whether there exist dominant patterns of similarities between institutions. Therefore, to extract such patterns we derive the Minimal Spanning Tree (MST) representation of borrowing companies' balance-sheet similarities (see Mantegna and Stanley, 1999; Bonanno et al., 2003; Spelta and Araújo, 2012).

### 2.4. The Minimal Spanning Tree

To find out the MST representation of the system, we perform hierarchical clustering by applying the nearest neighbor method. At the initial step, we consider *N* clusters corresponding to the *N* institutions. Then, at each subsequent step, two clusters *l*_*i*_ and *l*_*j*_ are merged into a single cluster if:

$$d\left(l_{i},l_{j}\right)=\min\left\{d\left(l_{i},l_{j}\right)\right\}$$

with the distance between clusters being defined as:

$$d\left(l_{i},l_{j}\right)=\min\left\{d_{rq}\right\}$$

with *r* ∈ *l*_*i*_ and *q* ∈ *l*_*j*_. These operations are repeated until a single cluster emerges. This clustering process is also known as the single link method since one obtains the MST of a network. Given a connected graph, the corresponding MST is a tree of *N* − 1 edges that provides the minimum value of the sum of the edge distances. More specifically, the hierarchical clustering procedure takes *N* − 1 steps to be completed when the graph is composed by *N* nodes, and it exploits, at each step, a particular distance *d*_*i,j*_ ∈ **D** to merge two clusters into a single one.

In order to extract relevant information from the topology of the network for discriminating between borrowing companies, we compute different measures from complex network theory. In particular, the research in network theory has dedicated a huge effort to developing measures of interconnectedness, related to the detection of the most important player in a network. Moreover, beside investigating the importance each institution has in the network, we are also interested in assessing whether the network is characterized by a community structure and to exploit such feature. This topological characteristic indicates the presence of sets of companies usually defined as very dense sub-graphs, with few connections between them.

### 2.5. Network Measures

Various measures of centrality have been proposed in network theory such as the count of neighbors of a node has, i.e., the degree centrality, or measures based on the spectral properties of the graph (see Perra and Fortunato, 2008). These measures are feedback, also know as global, centrality measures and provide information on the position of each node relative to all other nodes. For our purposes we employ both families of centrality measures. In particular, for each node we compute the degree and strength centrality. The degree *k*_*i*_ of a vertex *i* with (*i* = 1, …, *N*) is the number of edges incident to it. More formally, let the binary representation of the network be $\hat{\text{D}}$ such that:

$${\hat{D}}_{ij}=\left\{ \begin{array}{l} \;if\;d_{ij}>0\\ \;otherwise \end{array}\right.$$

then, the degree a vertex *i* is:

(8) $$\begin{array}{r} k_{i}=\overset{N}{\underset{j=1}{\sum }}{\hat{\text{D}}}_{ij}. \end{array}$$

Similarly, the strength centrality measures the average distance of a node with respect to its neighbors. Formally the strength of vertex *i* is:

(9) $$\begin{array}{r} s_{i}=\overset{N}{\underset{j=1}{\sum }}{\text{D}}_{ij}. \end{array}$$

Moreover, since several studies have found the presence of sets of very dense sub-graphs, with few connections between them, as a result of similar patterns at the micro-level (see Pecora et al., 2016; Spelta et al., 2018), we also apply the Louvain Method to extract the community structure of the network (see Blondel et al., 2008). The identified communities maximize system's modularity, a measure that quantifies the strength of the division of the system into communities of densely interconnected nodes that are only sparsely connected with the rest of the system (see Newman, 2006). The modularity of our system is:

(10) $$\begin{array}{r} Q=\frac{1}{2m}\underset{i,j}{\sum }\left[D_{i,j}-\frac{s_{i}s_{i}}{2m}\right]\delta \left(c_{i},c_{i}\right) \end{array}$$

where *d*_*i,j*_ is the weight of the edge between nodes *i* and *j*, *s*_*i*_ is the sum of the weights of the edges attached to node *i*, *c*_*i*_ is the community to which node *i* belongs, δ(*u, v*) is equal to 1 when *u* = *v* and zero otherwise, and $m=\frac{1}{2}\underset{i,j}{\sum }D_{i,j}$. The final step of our model specification is to embed the obtained centrality measures as well as information on the community structure of the network, into a predictive model. We propose to extend Chinazzi and Reyes, who incorporate network measures in a linear regression model, to the credit scoring context (i.e., logistic regression, linear discriminant analysis, CART, and SVM).

## 3. Results

**Figure 1:**
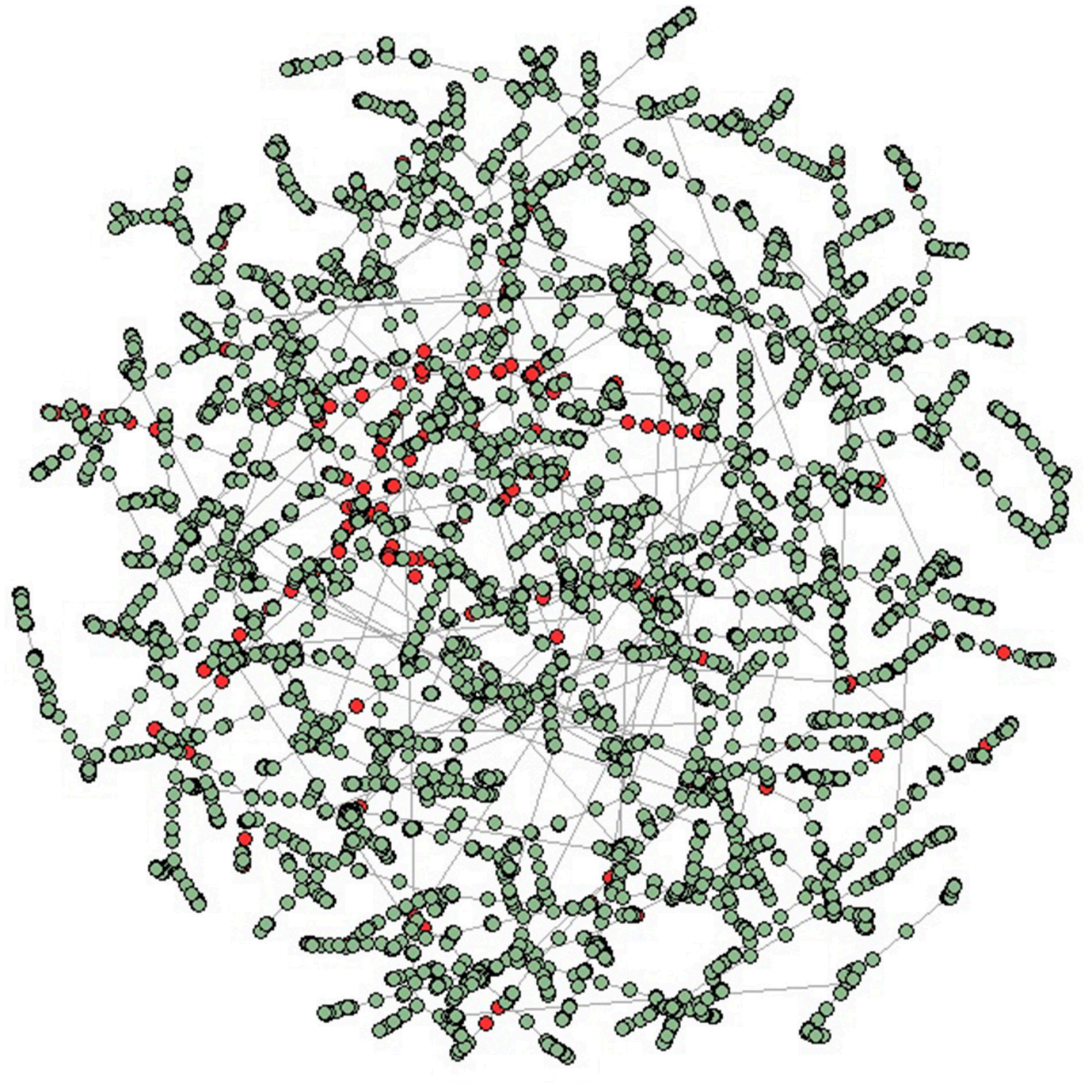
Minimal spanning tree representation of the borrowing companies networks. The tree has been obtained by using the standardized Euclidean distance between institutions features and the Kruskal algorithm. In the panel, nodes are colored according to their financial soundness, red nodes represent defaulted institutions while green nodes are associated with active companies.

This section is devoted to show the results of the analysis. First, we report the MST representation of the similarity network obtained from companies' feature distances. We show nodes colored according to their financial soundness, red nodes represent defaulted institutions while green nodes represent sound and active companies, see Figure 1. Notice how, defaulted institutions occupy precise portion of the network, namely, such companies belong to the leafs of the tree and form clusters. This, in other words, suggests those companies form communities.

Information concerning the community structure of the networks and the centrality measures are used to provide synthetic topological variables at the node level. Such variables are embedded into the credit scoring models to assess whether they contain relevant information useful for forecasting institutions default.

**Figure 2:**
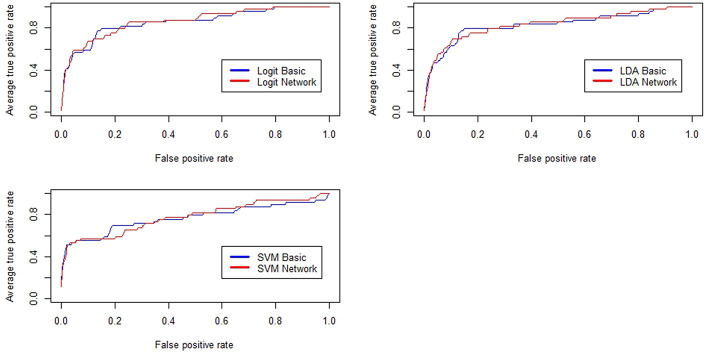
Receiver Operating Characteristic (ROC) curves for the baseline credit risk models and for the network-augmented models. In blue, we show the results related to the baseline models while in red we show the results related to the network-augmented models.

Figure 2 reports the results related to the performance of some of the models tested in the paper. Basically, the upper left panel shows the results from the logistic regression, the upper right panel encompasses the same information from the discriminant analysis while the bottom panel refers to the performance curves of the SVM classifier.

For sake of comparison, we have reported several measures of predictive utility so to show that, overall, the inclusion of topological information regarding similarity patterns among companies feature, increases the forecasting performance of various credit scoring models even when the data sets are imbalanced between the two classes (defaulted vs. active). Notice how, for most of the cases, red lines representing the performance of the models feeded with network measures lie above the blue lines representing baseline classifiers. Considering that graphically the improvements might not be fully visible, performance improvements for all the tested models are also reported in Table 2. The table summarizes the values of the measures employed to assess the predictive gain of the network-augmented credit scoring models. We report, the area under the ROC curve (AUC), the KS statistic, the Gini Index and the overall model accuracy (ACC).

**Table 2:** Summary Statistics of non-parametric analysis.

	**AUC**		**KS**		**Gini**		**Accuracy**	
	**Basic**	**Network**	**Basic**	**Network**	**Basic**	**Network**	**Basic**	**Network**
Logit	79.631	80.793	52	52	59.262	61.586	90.193	90.09661
LDA	77.759	79.16	51	52.8	55.518	58.32	90.122	89.98844
CART	67.973	67.973	35.5	35.946	35.946	35.5	90.832	90.82413
SVM	76.81	77.65	53.62	50	51	55.3	92.44444	92.22222

*Summary statistics of the non-parametric analysis. From the left to the right: area under the ROC curve (AUC), KS Statistic (KS), Gini Index (Gini), Model accuracy (Accuracy), and area under the Precision Curve (AUCPR). For each measure and for all the tested models we report the results obtained by the baseline scenario and for the network-augmented configurations*.

From the results collected in Table 2, it is clear that the inclusion of topological variables describing institutions centrality in the similarity networks and the community structure composing such networks increases the predictive performance of the methods used for credit scoring even if the forecasting gain obtained differ from model to model. In particular, we observe an increase of the predictive utility values for the logistic regression, the linear discriminate analysis and the SVM classifier once network parameters are added to the specification. Concerning the overall models accuracy, the ACC measure is less sensitive to the inclusion of topological variables with values between the baseline and network-augmented methods remaining quite similar across all models. Even though the increases in predictive utility across models are not very large, it might make significant difference for P2P lending platforms. Furthermore, we also notice that the predictive utility of the CART model does not change with the inclusion of the community and network parameters in the models specification. Future research may concern dealing with unbalanced samples (as in Calabrese and Giudici, 2015) and/or with multiple data sourrces (as in Figini and Giudici, 2011).

## 4. Conclusion

FinTech services, such as peer-to-peer lending platforms, are becoming part of the everyday life. Such new technologies can increase financial inclusion, but they can bring the cost of an increase credit risks. To cope with such risk, fintech risk management becomes a central point of interest for regulators and supervisors, to protect consumers and preserve financial stability. In this work we have shown that topological information embedded into similarity networks can be exploited to increase the predictive performance of credit scoring models usually applied by P2P lending companies. Topological information are summarized computing centrality measures and community detection. The forecasting gain obtained by the inclusion of these variables has been then measured by employing non-parametric statistics. Standard performance measures such as ROC, precision recall and accuracy reveal the usefulness of the proposed methodology to build an early-warning signal suitable for both policy makers and supervisors as well as for practitioners.

## Data Availability

All datasets generated for this study are included in the manuscript and/or the Supplementary Files.

## Author Contributions

It is the result of a joint work between the three authors in which, however, PG supervised the work and provided the necessary research framework. BH-M wrote sections Introduction, Credit Risk Models, Assessing Model Performance and Results. AS wrote sections The Distance Metric, The Minimal Spanning Tree, Network Measures, and Conclusion.

### Conflict of Interest Statement

The authors declare that the research was conducted in the absence of any commercial or financial relationships that could be construed as a potential conflict of interest.

## Appendix

### A. Financial Ratios

Since the data set is composed of ratios between financial and balance-sheet statements here we report the formulas employed to compute such ratios.

**Table A1:** Description of variables included in the dataset.

**ID**	**FORMULA**	**Type**	**ID**	**FORMULA**	**Type**
RATIO001	(Total assets - Shareholders Funds)/Shareholders Funds	Continuous	RATIO019	Interest paid/(Profit before taxes + Interest paid)	Continuous
RATIO002	(Long term debt + Loans)/Shareholders Funds	Continuous	RATIO027	EBITDA/interest paid	Continuous
RATIO003	Total assets/Total liabilties	Continuous	RATIO029	EBITDA/Operating revenues	Continuous
RATIO004	Current assets/Current liabilties	Continuous	RATIO030	EBITDA/Sales	Continuous
RATIO005	(Current assets - Current assets: stocks)/Current liabilties	Continuous	RATIO036	Constraint EBIT	Dichotomous
RATIO006	(Shareholders Funds + Non current liabilities)/Fixed assets	Continuous	RATIO037	Constraint PL before tax	Dichotomous
RATIO008	EBIT/interest paid	Continuous	RATIO039	Constraint Financial PL	Dichotomous
RATIO011	(Profit (loss) before tax + Interest paid)/Total assets	Continuous	RATIO040	Constraint P/L for period th EUR	Dichotomous
RATIO012	P/L after tax/Shareholders Funds	Continuous	DPO	Trade Payables/Operating revenues	Continuous
RATIO013	GROSS PROFIT/Operating revenues	Continuous	DSO	Trade Receivables/Operating revenues	Continuous
RATIO017	Operating revenues/Total assets	Continuous	DIO	Inventories/Operating revenues	Continuous
RATIO018	Sales/Total assets	Continuous	NACE	Industry classification on NACE code, 4 digits precision	Dichotomous
